# Fertility response in postpartum anoestrus buffaloes *(Bubalus bubalis)* using modified Ovsynch based timed insemination protocols

**DOI:** 10.14202/vetworld.2015.316-319

**Published:** 2015-03-12

**Authors:** K. K. Gupta, S. N. Shukla, P. Inwati, O. P. Shrivastava

**Affiliations:** Department of Veterinary Gynaecology and Obstetrics, College of Veterinary Science & Animal Husbandry, Nanaji Deshmukh Veterinary Science University, Jabalpur, Madhya Pradesh, India

**Keywords:** anoestrus buffaloes, fertility, insulin, ovsynch synchronization, timed artificial insemination

## Abstract

**Background and Aim:**

Suboestrus constitutes the largest factor responsible for poor reproductive efficiency in buffaloes. Therefore, oestrus synchronization (OS) and fixed-time artificial insemination (AI) is considered an alternative approach to enhance reproductive efficiency in buffaloes. Thus, the present study was carried out to study the efficacy of modified Ovsynch protocol with fixed time insemination in post-partum anoestrus buffaloes.

**Materials and Methods:**

Total 50 post-partum anoestrus dairy buffaloes were selected and randomly divided into 5 Groups, each comprising ten animals (n=10). Animals of Group I received buserelin acetate 10 μg(GnRH) at day 0 and 9, and prostaglandin F2α (PGF_2_α) at day 7; in Group II similar to Group I except double dose of Gn RH (20 μg) at day 0; in Group III, similar to Group I plus additional administration of insulin on day 0, 1 and 2; in Group IV, similar to Group II plus additional administration of insulin on day 0,1 and 2; in Group V similar to Group I except replacement of first Gn RH with insulin on day 0,1 and 2. Animal of all groups were inseminated at fixed time using frozen semen at 60 h and 72 h after PGF_2_α administration and confirmed for pregnancy at day 60 post-insemination.

**Results:**

The results revealed satisfactory and comparable synchronization of oestrus (60-80%) and conception rate (20-40%) among the various treatment groups in the present study. The better synchronization was observed in modified protocols. However, non-significantly higher conception rate was recorded in modified Ovsynch protocol (40% each in Group II-V) as compared to 20% in Group-I (p>0.05).

**Conclusion:**

In this study comparatively higher OS and conception following insulin modified Ovsynch based timed AI protocols in post-partum anoestrus buffaloes was found satisfactory and comparable.

## Introduction

Oestrus synchronization (OS) and fixed-time artificial insemination (TAI) has been considered a better alternative to overcome the problems of sub-oestrus in buffaloes. Ovsynch is one of the OS protocol consisting two injections of gonadotropic releasing hormone (GnRH) combined with single administration of prostaglandin F_2_ alpha (PGF_2_α) and used in cyclic buffaloes for synchronization of ovulation and TAI [1,2]. Efficacy of the Ovsynch is depended on the stage of follicular development at the time of initial GnRH injection [3]. The main advantage of Ovsynch protocol is OS that can be done in buffaloes at all stages of the cycle [1]. However, fertility is not promising with Ovsynch especially in anoestrus buffaloes.

Insulin has been used for the management of anoestrus in buffaloes [4,5]. Insulin stimulates the release of GnRH from hypothalamus and release of luteinizing hormone (LH) from pituitary [6,7]. Administration of insulin also increases peripheral as well as intra follicular insulin-like growth factor (IGF-I) levels [8]. Better oestrus induction and conception was recorded in insulin modified protocols as compared to the original Ovsynch in anoestrus buffaloes [5]. However, literature is lacking regarding effect of insulin modified Ovsynch protocol on OS and conception using fixed time insemination in anoestrus buffaloes.

Thus, the present study was carried out to study the efficacy of modified Ovsynch protocol with fixed time insemination in post-partum anoestrus buffaloes.

## Materials and Methods

### Ethical approval

The experiments on animals including all procedures of this study were approved by Institutional Animal Ethics Committee.

### Location and place of work

Post-partum buffalo herds maintained at livestock farm, Adhartal of Veterinary College and organized dairy farms in Pariyat area of Jabalpur District (Madhya Pradesh) were used for this study.

### Drugs and biologicals

GnRH - Buserelin acetate 0.0042 mg equivalent to 0.004 mg Buserelin per ml. PGF_2_α - Cloprostenol sodium 263 µg equivalent to Cloprostenol 250 µg per ml. Insulin - long acting biphasic Human Insulin (r-DNA origin) 30/70, 100 IU per ml. Frozen semen - frozen semen of Murrah buffalo in French mini straws supplied by central semen station, Bhadbhada, Bhopal, Madhya Pradesh was used for AI.

### Selection of experimental animals

The study was conducted in fifty apparently healthy dairy buffaloes maintained at organized dairy farms with the history of anoestrus since 4 month or more after calving. The selection of animals was done based on history of anoestrus and gynaecological examinations of genitalia at 10 days interval. Animals having clinically smooth ovaries were selected for the experiment.

### Experimental design

The selected buffaloes were randomly divided into 5 Groups, each comprising 10 animals (n=10). Animal of Group I (Ovsynch protocol) were administered intramuscularly buserelin acetate (10 μg) at day 0 followed by cloprostenol (500 μg) on day 7, and 2^nd^ dose of GnRH (10 µg) on day 9. In Group II (Modified Ovsynch protocol - I), intramuscularly buserelin acetate (20 μg) at day 0 followed by cloprostenol (500 μg) on day 7, and 2^nd^ dose of GnRH (10 µg) on day 9. In Group III (Modified Ovsynch protocol - II), buserelin acetate (10 μg) intramuscularly at day 0 followed by Insulin (@ 0.25 lU/kg body weight [b. wt,]) subcutaneously on day 0, 1 and 2 and Cloprostenol (500 μg) intramuscularly at day 7, and second dose of GnRH (10 µg) on day 9. In Group IV (Modified Ovsynch protocol - III), buserelin acetate (20 μg) intramuscularly at day 0, followed by Insulin (@ 0.25 lU/Kg b. wt,) subcutaneously on day 0, 1 and 2, then Cloprostenol (500 μg) was administered intramuscularly on day 7, and GnRH (10 µg) on day 9. In Group V (Modified Ovsynch protocol – IV), Insulin @ 0.25 IU/Kg b.wt, subcutaneously on day 0, 1 and 2 followed by Cloprostenol (500 μg) intramuscularly on day 7, and GnRH (10 µg) on day 9.

### Monitoring of animals for fertility response

All the animals were inseminated at 60 h and 72 h after PGF_2_α injection using frozen semen. Fertility response in terms of OS and conception rate was studied. The synchronization rate was assessed by visual observation for oestrus signs and per rectal examination at the time of AI as per the criteria given in Table-1. All animals were examined per rectally for confirmation of pregnancy at 60 days post-insemination and conception rate was calculated.

**Table-1:** Criteria used for grading of OS response in buffaloes.

Parameters during TAI	OS response				

	Excellent	Good	Fair	Poor	Nil
Vulvar lips swollen and edematous	Fully	Partially	Less	Less	Wrinkled
Vulvar mucus membrane moist and congested	Fully	Moist and Less Congestion	Moist and Pale	Slight Moist and Pale	Dry and Pale
Cervico-vaginal mucus discharge	Copious	Scanty	Not observed	Not observed	Not observed
Lubrication of vaginal lumen	Excellent	Good	Slight	Slight	Nil
External OS of cervix opened	Completely	Completely	Partially	Partially	Closed

TAI=Time artificial insemination, OS=Oestrus synchronization

### Statistical analysis

Results of synchronization and conception rate were expressed in percentage, and the obtained data were analyzed by Chi-square test using software SYSTAT^®^ version-11.0.

## Results

Fertility response in terms of OS and conception rate were recorded 60 and 20% in Group-I; 70 and 40% in Group-II; 80 and 40% each in Group-III-V, respectively in the present study. The results show similar and comparatively higher synchronization in Group III-V (80% in each) followed by Group-II (70%) and Group-I (60%). All the animals were inseminated at fixed time following treatment protocol, and none of the animals in any group exhibited signs of oestrus during the treatment.

Further, based on the intensity of oestrus signs observed during fixed time insemination, the OS was graded as excellent, good, fair, poor and nil as presented in Table-2. The analysis of results revealed better synchronization in insulin modified protocols (Group-III-V) as compared to the other groups.

**Table-2:** Grading of OS response using Ovsynch based protocol.

Groups (n=10)	OS response [n (%)]				

	Excellent	Good	Fair	Poor	Nil
I	2 (20)	1 (10)	2 (20)	1 (10)	4 (40)
II	3 (30)	2 (20)	1 (10)	1 (10)	3 (30)
III	4 (40)	2 (20)	1 (10)	1 (10)	2 (20)
IV	4 (40)	1 (10)	2 (20)	1 (10)	2 (20)
V	5 (50)	1 (10)	1 10	1 (10)	2 (20)

OS=Oestrus synchronization

The conception rate after fixed time insemination was recorded higher and similar in Group-II-V (40% in each) as compared to Group-I (20%). The results also revealed statistically nonsignificant higher conception in insulin modified Ovsynch protocols where the intensity of estrus was better during fixed time insemination (p>0.05). Similarly, non-significantly higher conception was recorded in Ovsynch protocol using 20 µg buserelin at day 0 than 10 µg (40 vs. 20%) and in the modified Ovsynch protocol (Group-V) where insulin completely replaced the first GnRH at day 0 (p>0.05).

## Discussion

The results of fertility response revealed similar and comparatively higher (p>0.05) synchronization in insulin modified protocols (80% each in Group III-V) followed by modified protocol without insulin (70% in Group II) and Ovsynch protocol (60% in Group I). The higher and satisfactory results of OS in insulin modified Ovsynch protocols are comparable to other findings [2] where synchronization was reported 83.33-100% using insulin modified Ovsynch protocol in post-partum buffaloes. The higher and satisfactory ovulation (88.8-90%) was also reported using Ovsynch protocol by different workers [1,9,10] however; in another study ovulation was found 60% for acyclic buffaloes and 81% for cyclic buffaloes during summer season [11].

The beneficial effects of insulin on resumption of ovarian cyclicity and fertility in buffaloes may be due to its effects on folliculogenesis and steroidogenesis [12,13]. Insulin enhances growth and proliferation of granulosa, theca and luteal cells in the ovary [13,14]. This stimulate folliculogenesis either acting through specific insulin receptor and IGF-1 or both type of the receptors. Insulin also induces LH pulse secretion and thus maturation of follicles [7]. Exogenous application of insulin along with gonadotropins resulted in greater diameter of a large follicle and increased the level of IGF-1 in follicular fluid [8]. IGFs stimulate growth and maturation of follicles through granulosa cell proliferation, steroidogenesis as well as inhibin and activin synthesis in FSH dependent manner [15,16]. Beneficial effects of insulin alone and in combinations using GnRH and PMSG in anoestrus buffaloes were also reported [4,5] which supports the present study.

The conception rate following TAI was recorded higher and similar to modified Ovsynch protocols (40% in each in Group II-V) as compared to the Ovsynch protocol using 10 µg GnRH at day 0 and 9 (G I, 20%) in anoestrus buffaloes (p>0.05). The results of comparatively lower conception rate in different protocols of the present study (20-40%) are comparable to the findings of several workers [1,9,10] who used GnRH- PGF_2_-alpha - GnRH (G-P-G) protocol with fixed time insemination. However, in another study using G-P-G protocol and inseminated at 16-20 h after the second dose of GnRH found 18% conception in cyclic buffaloes whereas, none of the non-cyclic buffalo conceived during the summer season [11]. It was suggested that remarkably low conception rate in buffaloes may be due to early ovulation and sub-functional corpus luteum [9]. Presence of large follicles at the beginning of Ovsynch protocol is found to be a determining factor for successful synchronization of ovulation and high conception in buffaloes [3]. However, ovulation occurs earlier and over all wider range of time in the non-cyclic buffaloes, ranging 12-36 h with an average 26±4.8 h as compared to the cyclic buffaloes which may be the reason of nil conception during summer season [11].

The literature is lacking regarding the use of proposed modified TAI Ovsynch protocol with insulin in post-partum anoestrus buffaloes. However, highest conception in postpartum buffaloes using insulin modified Ovsynch protocol (66.66%) was recorded where second dose of GnRH was completely replaced by insulin on day 8, 9 and 10 followed by protocol using half dose of day 9 GnRH plus additional administration of insulin on day 8, 9 and 10 (58.33%); lowest in the protocol where additional insulin was administrated on day 8, 9 and 10 [2]. In his study, no effect on conception (50% in each) was found using half dose of day 9 GnRH in Ovsynch protocol.

Better conception (75-88.89%) was reported using such modified Ovsynch protocols with insulin in post-partum anoestrus buffaloes as compared to the present study [5]. Comparatively better results in his study may be due to breeding of buffaloes at induced oestrus by natural service, unlike the present study where TAI was performed using frozen semen without detection of oestrus. The use of insulin in the modified protocols may be one of the reasons for better conception as reported in cattle [17].

## Conclusion

Comparatively higher OS and conception following insulin modified Ovsynch based TAI protocols in post-partum anoestrus buffaloes were found satisfactory and comparable. Insulin is a non-steroidal metabolic hormone, cheap, easily available and has a beneficial effect in the modified Ovsynch based protocols thus can be an effective alternative to better fertility response in anoestrus buffaloes.

## Authors’ Contributions

KKG has done the experiments for his thesis research work. SNS has designed experiment and monitored all the research activities being a major advisor. PI has helped in experiments especially in treatments and AI. OPS has helped in thesis work as head of department. All authors participated in drafting and revision of the manuscript. All authors read and approved the final manuscript.
